# First report on isolation of *Listeria ivanovii* from a cat

**DOI:** 10.1186/s12917-025-05007-0

**Published:** 2025-09-24

**Authors:** Dorota Chrobak-Chmiel, Magdalena Kizerwetter-Świda, Ewelina Kwiecień, Ilona Stefańska, Rafał Sapierzyński, Rafał Nejfeld, Dorota Żabicka, Małgorzata Dziekiewicz-Mrugasiewicz, Magdalena Rzewuska

**Affiliations:** 1 Department of Preclinical Sciences, Institute of Veterinary Medicine, Warsaw University of Life Sciences-SGGW, Ciszewskiego 8, Warsaw, 02-786 Poland; 2 Department of Pathology and Veterinary Diagnostics, Institute of Veterinary Medicine, Warsaw University of Life Sciences-SGGW, Nowoursynowska 166, Warsaw, 02-787 Poland; 3 Department of Epidemiology and Clinical Microbiology, National Medicines Institute, Chełmska 30/34, Warsaw, 00-725 Poland; 4 Department of Large Animals with Clinic, Institute of Veterinary Medicine, Warsaw University of Life Sciences-SGGW, Nowoursynowska 100, Warsaw, 02-797 Poland

**Keywords:** *Listeria Ivanovii*, Cat, Tissue samples, Infection

## Abstract

**Background:**

Several *Listeria* species cause listeriosis. *Listeria monocytogenes* is responsible for the majority of clinical cases in animals. A less frequently isolated species is *Listeria ivanovii*, which causes infections mainly in ruminants, most often associated with reproductive disorders, such as abortions, stillbirths, or neonatal mortality. Sporadic infections with this microorganism have been described in other animal species; however, no literature data, to our knowledge, describe the infection of *L. ivanovii* in cats.

**Case presentation:**

*L*. *ivanovii* was isolated from tissue samples collected from a kitten with symptoms of acute catarrhal gastroenteritis. Fragments of the liver, spleen, kidneys, and small intestine were collected post-mortem for microbiological examination. The isolated microorganism was identified as *L. ivanovii* based on its phenotypic properties and 16 S rRNA sequencing. The identification of subspecies *L. ivanovii* subsp. *ivanovii* was performed by comparative analysis of the *sigB* gene sequences.

**Conclusions:**

*Listeria ivanovii*, which is more commonly known as a ruminant pathogen, can also cause severe infections in cats. This is, to our knowledge, the first report on the isolation of this microorganism from internal organs in a cat. This finding underscores the importance of careful interpretation of isolation results when uncommon pathogens are involved, particularly in host species where their pathogenicity has not been previously documented.

## Background

Bacteria within the genus *Listeria* are Gram-positive, non-spore-forming, non-capsulated, facultatively anaerobic rods. These microorganisms are widely distributed in the environment, such as soil, water, and decaying organic matter. Moreover, *Listeria* species frequently contaminate various foods, including meats, milk, and cheese. Additionally, it contaminates animal feed, such as silage [1].

According to the List of Prokaryotic names with Standing in Nomenclature (LPSN), the *Listeria* genus consists of 29 species [2]. Only two *Listeria* species are considered clinically important and pathogenic, with *Listeria ivanovii* primarily affecting ruminants and *Listeria monocytogenes* affecting various animal species and humans [3–5].

*Listeria ivanovii* has been divided into two subspecies: *ivanovii* and *londoniensis*. They can be differentiated based on biochemical properties, such as ribose fermentation which is positive only for the subspecies *ivanovii* and N-acetyl-β-D-mannosamine fermentation, which is positive only for the subspecies *londoniensis*. Additionally, they can be distinguished based on their susceptibility to temperate phages (B025, B035, B054, and B056). Only the strains of *L. ivanovii* subsp. *londoniensis* seemed to be resistant to the bacteriophages mentioned above [1, 6]. However, it is strongly believed that among these two subspecies, *Listeria ivanovii* subsp. *ivanovii* is more virulent and responsible for most described cases of listeriosis in animals and humans [7, 8].

Most studies on the distribution of *L. ivanovii* in the environment indicate its presence in fecal samples originating from various animal species [9–16]. On the other hand, infections caused by *L. ivanovii* are primarily observed in small ruminants and cattle, where they can result in septicemic disease with enteritis, neonatal sepsis, fetal death, stillbirth, and premature births [17, 18]. It is believed that *L. ivanovii* exhibits host tropism toward ruminants and rodents, with much lower virulence for humans compared to *L. monocytogenes*. However, it can occasionally cause infections in humans, resulting in bacteremia in immunocompromised individuals and stillbirths and abortions in pregnant women [1, 19]. Nevertheless, this species has not been previously described to be isolated from companion animals, including cats, and this is, to our knowledge, the first report of *L. ivanovii* isolated from tissue samples collected from a kitten.

## Case presentation

**Fig. 1:**
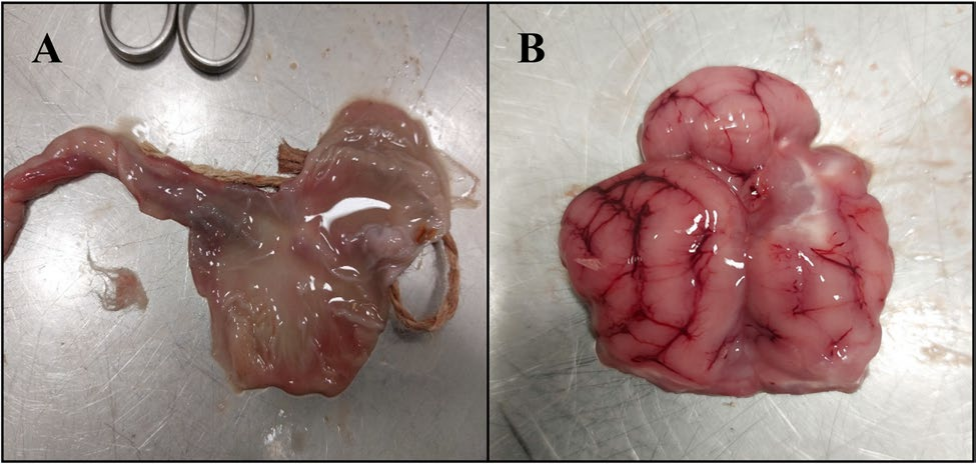
**A**- Acute catarrhal gastritis in the diagnosed kitten; **B**- Brain edema in the diagnosed kitten

A two-month-old female Maine Coon cat, which died with symptoms of acute catarrhal gastroenteritis, was delivered to the Department of Pathology and Veterinary Diagnostics, Institute of Veterinary Medicine, Warsaw University of Life Sciences for a post-mortem examination. Two other kittens from this litter died earlier with similar symptoms, but they were not diagnosed. The necropsy revealed signs of a wasting process (emaciation, loss of subcutaneous and visceral fat tissue, pallor of the mucous membranes), mild ascites, acute catarrhal gastroenteritis, blood stasis in internal organs, brain edema, and pulmonary edema (Fig. 1A and B). Tissue samples of the liver, spleen, kidneys, and a fragment of the small intestine were collected for microbiological examination.

**Fig. 2:**
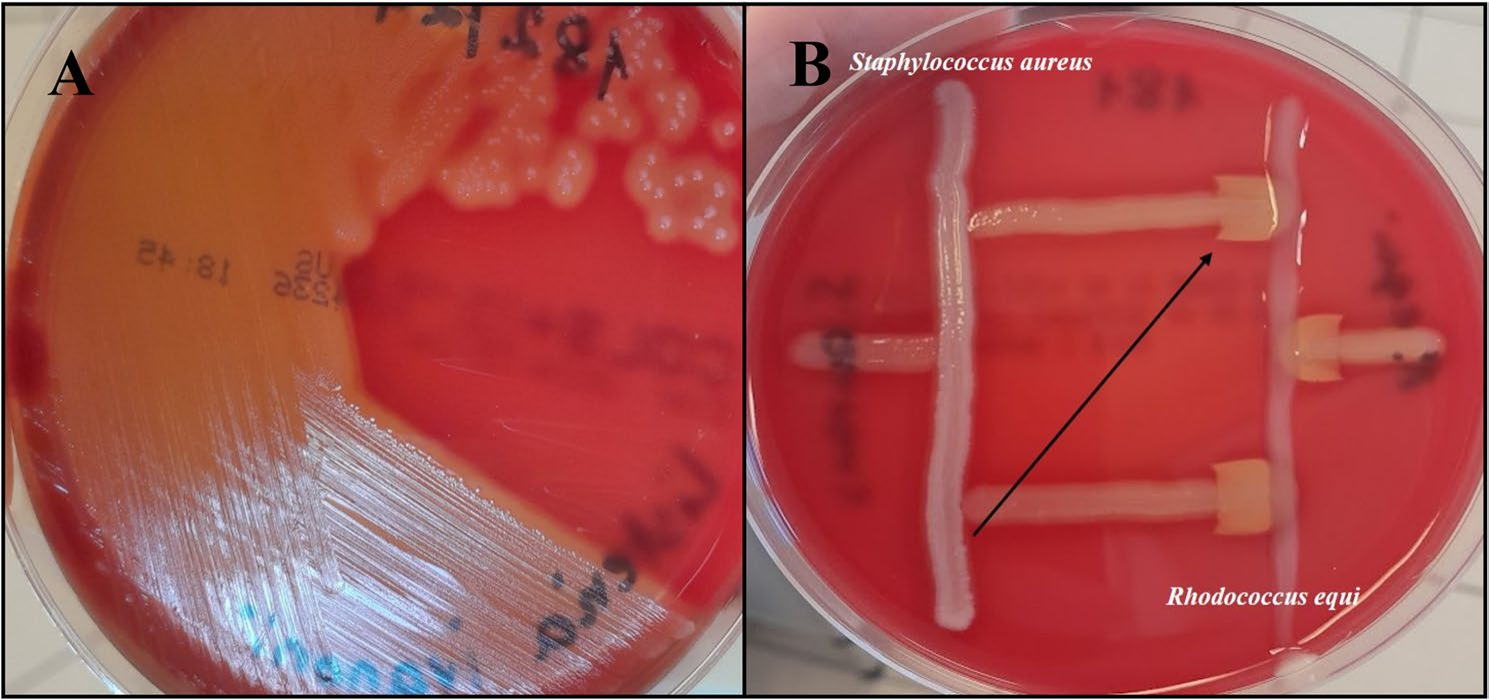
**A**- Colony morphology on Columbia blood agar after 48 h of incubation; **B**- CAMP test and enhancement of hemolysis by the tested *L. ivanovii* isolate with *Rhodococcus equi*

Clinical material was cultured on Columbia Agar plates with 5% sheep blood (Graso Biotech, Poland) and MacConkey Agar (Graso Biotech). Incubation was carried out in aerobic conditions and a 5% CO_2_ atmosphere at 35 °C. After 48 h of incubation, no growth was observed on MacConkey Agar, while on blood agar plates the presence of large numbers of medium-sized, white, shiny colonies surrounded by a wide zone of β-hemolysis was detected, and no other colonies were found (Fig. 2A). These colonies were isolated from all cultured tissue samples. Subsequently, the isolated microorganism was subjected to a routine bacteriological identification. The morphology of bacterial cells was assessed using the standard Gram staining method, and Gram-positive, short, regular rods were observed under the light microscope (Eclipse E400, Nikon, Japan). The test for catalase production was positive. In the CAMP test, two reference strains were used: *Staphylococcus aureus* ATCC 25,923 and *Rhodococcus equi* ATCC 25,729. A positive result, visible as enhancement of hemolysis, was observed only with *R. equi* (Fig. 2B).

Genomic DNA for molecular tests was obtained using the GeneMATRIX Bacterial & Yeast Genomic DNA Purification Kit (EURx, Poland). The final species identification was based on a sequenced 1,174 bp nucleotide fragment of the gene encoding 16 S rRNA (GeneBank accession number: PV984500). The primer sequences used in this study were as described by Zielińska et al. [20]. The thermal cycling for the PCR included an initial step of 95 °C for 4 min, followed by 35 cycles of 95 °C for 30 s, 60 °C for 30 s, 72 °C for 50 s, and 72 °C for 5 min. Additionally, the *sigB* gene sequencing was performed (GeneBank accession number: PV978369) using a primer set and PCR conditions described by Alvarez-Ordóñez et al. [21]. GenBank BLAST analysis of the nucleotide sequence of the gene encoding 16 S rRNA showed 99.91% identity to the sequences of *L. ivanovii* deposited in the NCBI database. Further analysis of the *sigB* gene showed that the isolate belonged to *Listeria ivanovii* subsp. *ivanovii*. The 780 bp nucleotide sequence of the *sigB* gene had 100% identity to *Listeria ivanovii* subsp. *ivanovii* and 96.4% identity to *Listeria ivanovii* subsp. *londoniensis.*

## Discussion

*Listeria ivanovii* subsp. *ivanovii* is an intracellular bacterium widely distributed in nature, responsible for listeriosis in ruminants and humans [4]. There is no data on the presence of this bacterium in pet animals, to our knowledge, this is the first report on the isolation of *L. ivanovii* from a cat. In general, there are fewer studies describing the pathogenicity and occurrence of *L. ivanovii* in animals and the environment compared to *L. monocytogenes.*

Studies conducted by Cao et al. [13] revealed the presence of *L. ivanovii* subsp. *ivanovii* and *L. ivanovii* subsp. *londoniensis* in 26 (3.7%) out of 702 fecal samples collected from 25 different species of wild rodents. The authors suggested that the wild rodents could act as the long-term host for *L. ivanovii*. This is in line with the study of Wang et al., who identified *L. ivanovii* in seven (20.6%) out of 341 intestinal fecal samples of rodent origin [12]. In addition to *L. ivanovii*, it is worth mentioning that both studies confirmed the presence of other *Listeria* species - namely: *L. monocytogenes*, *L. innocua*, *L. fleischmannii*, and *L. floridensis* – in fecal samples collected from rodents. In Belgium, Bauwens et al. [9] detected *L. ivanovii* in the feces of red ruffed lemur (*Lemur variegatus rubra*) and Grauer’s gorilla (*Gorilla gorilla graueri*) kept in Antwerp Zoo. Additionally, Sarangi and Panda [11] reported the presence of *L. ivanovii* in a fecal sample collected from a leopard (*Panthera pardus*). In Poland, this bacterium was also isolated from a red fox (*Vulpes vulpes*) rectal swab [22].

The presence of *L. ivanovii* was confirmed in cloacal swabs collected from birds, including chickens, pigeons, ducks, and turkeys. Furthermore, this bacterium was found to contaminate frozen chicken breast fillets [15]. The study conducted by Abuhatab et al. [15] highlights that birds are carriers of *L. ivanovii*; therefore, further studies are needed to confirm whether these animals can serve as a source of *L. ivanovii* in the environment.

In this study, the source of infection remains unknown. There is no information on the method of feeding and the type of food that was given to the kittens in a litter. It is known that other kittens within this litter died, but they were not diagnosed. Thus, it is not clear why *L. ivanovii* was present in the cat’s internal organs, but if this bacterium contaminates poultry meat, then such raw food may be a potential source of infection for carnivores, including cats. Additionally, shedding of *Listeria* in the rodent feces could contaminate food products or food-processing environments by direct or indirect transmission paths.

*L. ivanovii* was detected in the river and farm water. This suggests that contaminated water may also pose a risk of transmission of this bacterium to animals [14].

Palacios-Gorba et al. [23] identified wild animals, such as deer and wild boars, as potential reservoirs of *L. ivanovii*, which were detected in their tonsil samples. Moreover, it was shown that *L. ivanovii* was present in cattle, mainly in the udders. Single isolates were found in tonsils and feces [14, 16]. Furthermore, other studies indicate the presence of *L. ivanovii* in mastitic milk samples obtained from cattle, buffalo, and goats, suggesting this bacterium’s involvement in udder infections [1, 10].

As was mentioned before, there are only sparse data on the pathogenicity of *L. ivanovii*, however, unlike *L. monocytogenes*, which commonly induces meningoencephalitis, *L. ivanovii* is known not to cause such manifestations in ruminants. Its impact on humans is sporadic, with cases generally restricted to gastroenteritis, bacteremia in immunocompromised individuals, or fetal loss during pregnancy [24]. It should be emphasized that *L. ivanovii* exhibits a dynamic and complex array of virulence factors, highlighting its evolutionary adaptability [1]. Prominent among these is sphingomyelinase C (SmcL), encoded by the *Listeria* Pathogenicity Island-2 (LIPI-2), which disrupts host cell membranes and facilitates tissue invasion and immune evasion [25]. Ivanolysin O, a pore-forming hemolysin analogous to listeriolysin O in *L. monocytogenes*, supports escape from the phagolysosome into the cytoplasm, allowing intracellular survival and replication [1]. The phospholipase SmlC mediates a characteristic shovel-shaped “CAMP-like” response with *Rhodococcus equi*, which is used for phenotypic identification of *L. ivanovii*. It was suggested that SmlC contributes to host tropism as it effectively lyses sheep but not horse erythrocytes, which contain less sphingomyelin [1, 26]. Intriguingly, the regulation of virulence factors in *L. ivanovii* differs from that in *L. monocytogenes*. Both species rely on PrfA as a central transcriptional regulator, yet *L. ivanovii* demonstrates strain-specific variations in how these factors are expressed. These differences allow *L. ivanovii* to adapt its pathogenic potential to specific environmental and host contexts, enhancing its survival and ability to cause a disease [4].

Although listeriosis is rarely reported in cats, a few documented cases of *L. monocytogenes* infection, involving internal organs, lymphatic and serosal structures, suggest that felines are not resistant to infections by members of the *Listeria* genus [27, 28]. Therefore, our report confirms the susceptibility of cats to these bacteria and may represent an underrecognized route of *L. ivanovii* transmission in companion animals. This also suggests that these bacteria can cross species barriers and infect a broader range of hosts, regardless of their usual host specificity.

To our knowledge, no studies described infections caused by *L. ivanovii* in companion animals, including cats. Nevertheless, this bacterium can cause serious infections and can be isolated from internal organs, as confirmed by Kimpe et al. [29], who identified this microorganism in nodulated liver tissue from a septicaemic chinchilla (*Chinchilla lanigera*).

## Conclusion

*Listeria ivanovii* can be an etiological agent of severe infections in cats. This is the first report on the isolation of this microorganism from a companion animal.

## Data Availability

The datasets used and/or analysed during the current study are available from the corresponding author on reasonable request.

## Abbreviations

LPSN: List of Prokaryotic names with Standing in Nomenclature
CAMP test: Christie-Atkins-Munch-Petersen test
ATCC: American Type Culture Collection
NCBI: The National Center for Biotechnology Information
